# Inhaler sustainability in asthma and COPD care: a systematic review

**DOI:** 10.1136/bmjopen-2024-098052

**Published:** 2025-07-25

**Authors:** Adeola Ayodotun Onasanya, Yaser Haider, Grace Peaston, Agnieszka Ignatowicz, Alice M Turner

**Affiliations:** 1 Department of Applied Health Sciences, University of Birmingham, Birmingham, UK; 2 University of Birmingham Medical School, Birmingham, UK; 3 University Hospitals Birmingham NHS Foundation Trust, Birmingham, UK

**Keywords:** Asthma, Systematic Review, PUBLIC HEALTH

## Abstract

**Abstract:**

**Objective:**

To evaluate inhaler sustainability in asthma and Chronic Obstructive Pulmomary Disease (COPD) by analysing how inhaler design typology, prescribing and usage patterns, disposal and recycling practices influence human health and environmental outcomes, using a People-Process-Product (PPP) framework to identify actionable opportunities for improvement.

**Design:**

A systematic review was conducted in May 2024, with reporting structured around the PPP framework using narrative synthesis.

**Data sources:**

MEDLINE, Scopus, Cochrane Library and relevant grey literature were searched for publications over the period from April 2014 to April 2024.

**Eligibility criteria:**

Studies were included if published between 2014 and 2024, involved patients with asthma or COPD and healthcare professionals and specifically examined aspects of inhaler sustainability, including patient behaviours, healthcare provider prescribing practices and environmental impacts.

**Data extraction and synthesis:**

Two independent reviewers screened and extracted data from 63 studies. Due to diverse methodologies, quality assessment focused on research design robustness, completeness of outcome reporting and potential biases. Findings were synthesised narratively to address each research question using the PPP framework.

**Results:**

33% of included studies focused on two or more domains of the PPP framework as both primary and/or secondary outcomes. Studies mapped to the ‘People’ domain (n=34) showed limited awareness among patients and clinicians regarding the environmental impact of inhaler prescription patterns, use patterns and disposal methods, with over 75% of patients discarding inhalers in household waste. In the ‘Process’ domain (n=11), switching from pressurised metered-dose inhalers (pMDIs) to dry powder inhalers (DPIs) or soft mist inhalers (SMIs) was associated with improved inhaler adherence and asthma control, though uptake of new inhalers was influenced by patients’ prior experience, competence, proficiency and perceived usability. The ‘Product’ domain (n=41) showed that DPIs and SMIs consistently had lower carbon footprints than pMDIs, with short-acting beta-agonists (SABAs) pMDIs having the highest emissions due to prescription, use patterns and disposal.

**Conclusions:**

Improving patient education on sustainable inhaler use and disposal and providing healthcare professionals with focused training on low-carbon prescribing are critical steps towards achieving significant clinical benefits and supporting environmental sustainability in asthma and COPD management.

**PROSPERO registration number:**

CRD42024541927.

**STRENGTHS AND LIMITATIONS OF THIS STUDY**

- The review systematically identified key gaps in inhaler sustainability and developed practical recommendations to support environmental sustainability in asthma and COPD management.
- Applying the People-Process-Product framework enabled a structured synthesis of how patient and prescriber behaviour, prescribing processes and inhaler design characteristics affect inhaler sustainability.
- The methodological and outcome heterogeneity of the included studies prevented a meta-analysis, limiting the ability to estimate overall effect sizes.
- Limiting the review to English-language studies limits the comprehensiveness of the evidence base.

## Introduction

 Inhalers have transformed the treatment of respiratory diseases by providing an effective means of delivering medication directly to the lungs and are essential for millions of patients worldwide.^1^ Inhalers can be categorised based on device type or medication content. There are 3 types of inhaler devices: pressurised metered-dose inhalers (pMDIs), dry powder inhalers (DPIs) and soft mist inhalers (SMIs). In the UK, pMDIs are the most commonly prescribed inhaler device,^2^ contributing to climate change primarily through two key pathways: the use of propellant gases and associated life cycle emissions.^3^ Inhalers account for approximately 3% of the overall carbon footprint of the National Health Service (NHS).^4^ Consequently, transitioning to inhalers with a reduced global warming potential (GWP) could yield clinical, cost and environmental benefits.^5^

Inhalers can also be classified based on the drug category of the medication content. Common classes include short-acting beta-agonists (SABAs), short-acting muscarinic antagonists, inhaled corticosteroids (ICS), long-acting beta-agonists (LABAs), long-acting muscarinic antagonists (LAMAs) and combination medications containing 2–3 classes of drugs. Of these medication classes, SABA-containing inhalers have the greatest environmental impact due to their frequency of usage as rescue inhalers.^5 6^

Inhalers, while essential for managing respiratory conditions, also contribute to environmental harm, which can, in turn, affect human health.^7^ The environmental impact, driven by the emissions from propellants and waste generated by inhaler use, can exacerbate respiratory conditions, creating a cyclical relationship where increased inhaler use further harms the environment.^7 8^

To address this, there is a need for a deeper understanding of the complex relationship between human and environmental health as it relates to the inhaler. However, current research has not comprehensively assessed the combined human and environmental effects of inhalers in the asthma and Chronic Obstructive Pulmomary Disease (COPD) care pathway. Addressing this gap requires an understanding of the interaction between people, inhaler components and care processes in asthma and COPD care and making actionable recommendations tailored to specific stakeholders and processes related to the prescription, use and disposal of inhalers. As global efforts to reduce carbon emissions and promote sustainable practices intensify, there is a growing need to assess how inhalers can become more sustainable. Given the widespread reliance on inhaler devices globally, evaluating their sustainability from various perspectives is essential.

**Figure 1:**
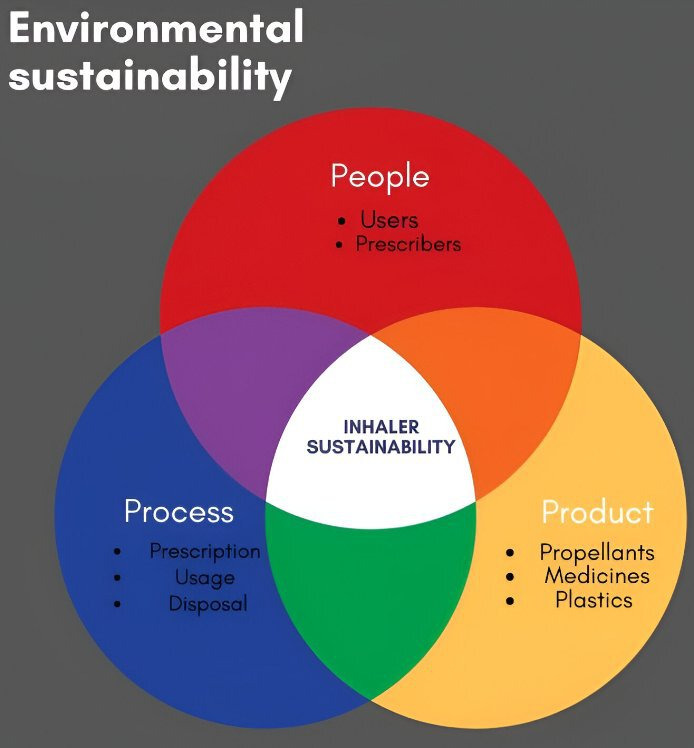
Components of inhaler sustainability in healthcare.

This systematic review aims to assess inhaler sustainability by focusing on the effect of inhaler design typology, prescription patterns, usage patterns, disposal and recycling on human health and environmental sustainability, while also identifying opportunities for improvement. To guide this exploration, we will assess inhaler sustainability through a tripartite framework focusing on people, processes and products, which highlights the interconnected nature of these elements in achieving sustainability outcomes (figure 1). The People-Process-Product (PPP) model, also known as the People, Process and Technology model, is a holistic model for process improvement known and used across industries,^9^ which can be applied to improving inhaler sustainability.

At the core of this framework are cyclical relationships between people, processes and products, which shape and influence each other over time. The ‘people’ perspective considers patients’ and healthcare providers’ awareness of inhaler sustainability, including factors influencing inhaler use, disposal habits and prescription. The ‘process’ aspect examines the transition to sustainable inhaler options, including the behaviour of patients and healthcare providers and its effects. Finally, the ‘product’ perspective evaluates the environmental impact of inhalers themselves, with a focus on the carbon footprint of the inhaler design and content.

Through this comprehensive multi-perspective analysis, the review aims to identify actionable recommendations for improving inhaler sustainability. The review addresses three interlinked questions that stem from the overarching framework:

1. What are the attitudes, actions and outcomes of patients towards the use, adherence to and disposal of sustainable inhaler options (device/medication)?
2. What are the attitudes, actions and outcomes of healthcare professionals in prescribing sustainable inhaler options (device/medication)?
3. What is the environmental impact of inhaler devices or medication use?

By addressing these questions, we aim to integrate the people, process and product perspectives, identifying both the opportunities and challenges for promoting inhaler sustainability.

## Methods

A systematic review was conducted in May 2024. Databases, such as MEDLINE, Scopus, Cochrane Library and the International Health Technology Assessment Database, were searched. Additional sources, including Google Scholar, BASE and RefSeek, were used to search for grey literature. The search strategy (online supplemental file 1) was developed after an initial scoping of the literature in April 2024. The systematic review was registered on the PROSPERO database under the registration number CRD42024541927.

### Study selection criteria

The inclusion criteria focused on studies published within the past decade that addressed inhaler sustainability through the people, process and product framework, including those on inhaler use, prescription, disposal and recycling, as well as patient and prescriber attitudes and behaviours. The review was limited to studies published from 2014 onwards to ensure relevance, following the release of the 2014 Intergovernmental Panel on Climate Change Synthesis Report^10^ and the UK Sustainable Development Strategy.^11^ These documents identified healthcare as both a contributor to climate change and a sector increasingly affected by its impact. Studies published after this period are more likely to reflect the heightened focus on environmental sustainability within health systems.

Eligible populations included patients with asthma or COPD and healthcare professionals caring for these patient groups. Studies were required to be published in English. Studies older than 10 years, non-academic sources such as news articles and reports unrelated to inhaler sustainability were excluded. Full criteria can be found in online supplemental file 2.

### Data extraction and quality assessment of studies

Two independent reviewers screened titles, abstracts and full texts, with a third reviewer resolving any discrepancies. Data were extracted using a standardised template, capturing study characteristics, population details, types of inhalers, sustainability interventions and outcome measures such as environmental impact and healthcare professional perspectives. To examine the sustainability constructs within the PPP framework, each study was coded to a predeveloped template for outcome measures by two reviewers. A third reviewer resolved any conflict. Thereafter, the study outcomes were coded into the PPP framework. Covidence software was used for data management.

Given the diverse study designs expected from this systematic review, no single standard risk-of-bias tool was applicable. We developed a quality assessment tool derived from fields contained in validated tools that generally addresses methodology, outcome data completeness, selective outcome reporting and other sources of bias (online supplemental file 3) and could be applied to all studies equally.

### Data synthesis and presentation

A narrative synthesis was conducted to integrate both qualitative and quantitative data. A narrative review of findings, considering the variability among studies, allowed us to explore the breadth of evidence and identify common trends or discrepancies without formal statistical pooling. Synthesis was framed within the ‘PPP’ framework to align findings with the research aims.

### Patient and public involvement

Patients and/or the public were not involved in the design, conduct, reporting or dissemination plans of this research.

## Results

### Overview of studies

**Figure 2:**
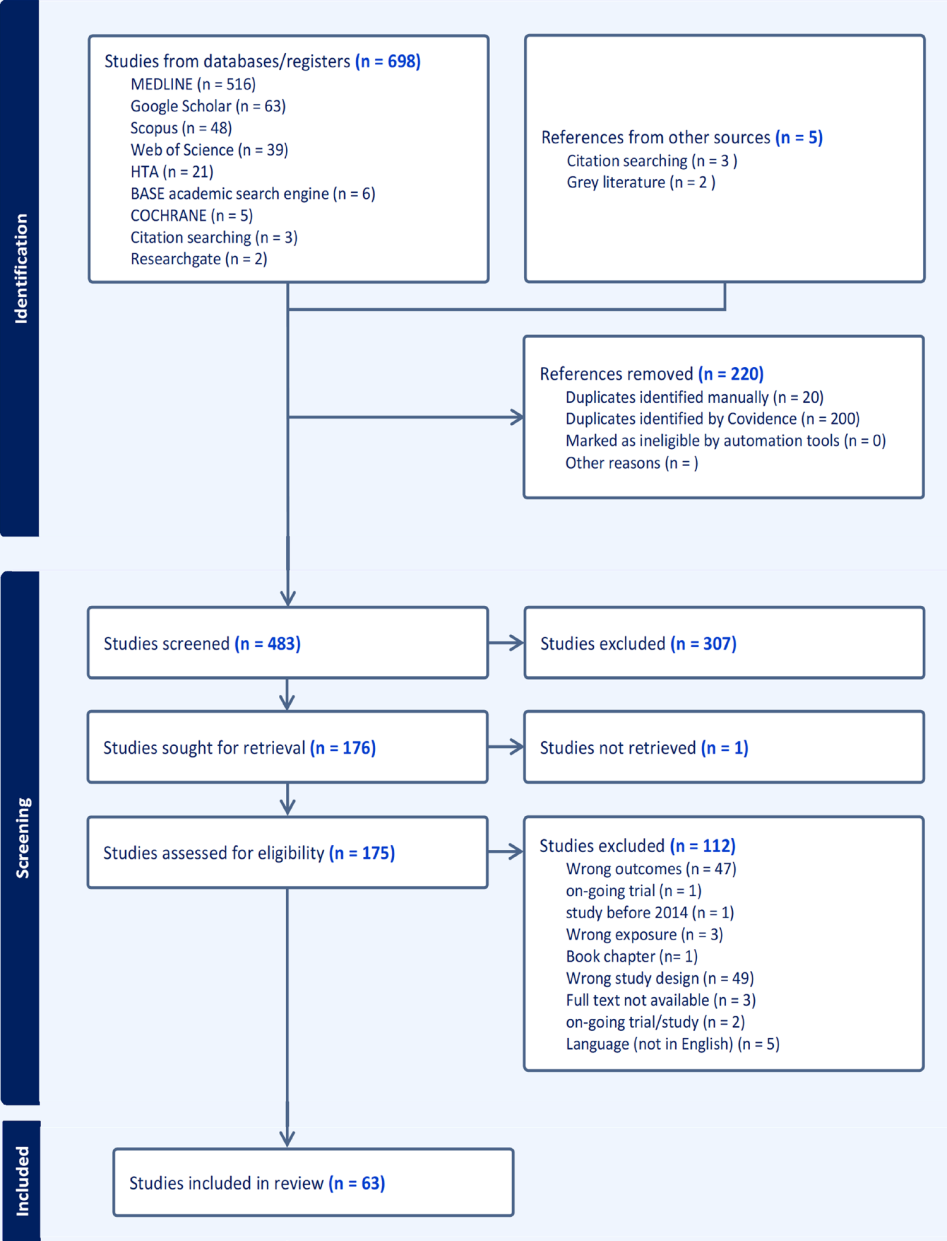
Preferred Reporting Items for Systematic Reviews and Meta-Analyses flowchart. HTA, Health Technology Assessment; n, number.

We identified 703 articles from our database searches, and 483 studies remained after removing duplicates. Screening narrowed this to 177 studies assessed for full-text eligibility, with 63 studies included in the final data extraction (figure 2). The results indicate a diverse range of studies, with approximately 46% (29/63) published in conference proceedings.^12^,^39^ 24 of the studies were primarily conducted in the UK,^528 30^,^33 35^ with 17 studies conducted in two or more countries.^614^,^16 18 24 26 27 47^ Industry sponsorship was a common theme, with 41% (26 studies) sponsored by pharmaceutical companies, particularly for the carbon footprint analysis.^16 7 14 16 18 25 27 44^,^47 49^ The quality of the studies varies significantly. Most studies used appropriate methodologies and reported comprehensive outcomes, but several studies, especially conference papers, showed incomplete or uncertain recruitment strategies and data reporting. Only 31 studies demonstrated sufficiently rigorous data analysis (online supplemental file 3). Journal articles had more consistent rigour in data analysis and reporting, although some show weaknesses in rigorous data analysis or outcome completeness.

**Figure 3:**
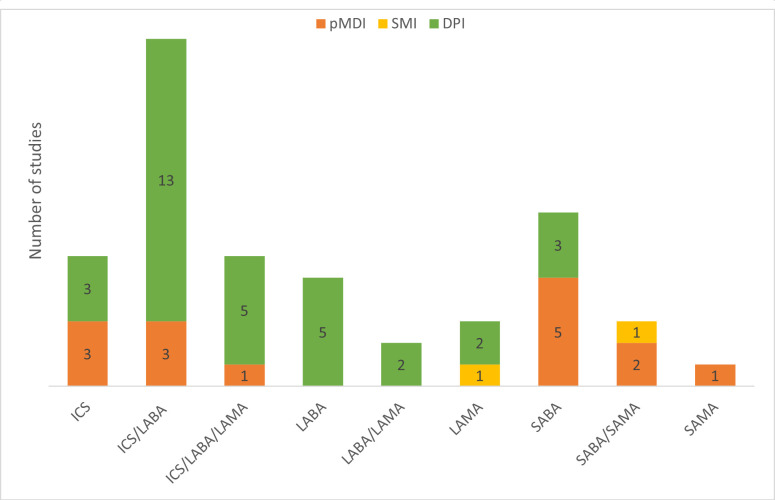
Inhaler devices by medication type. DPI, dry powder inhalers; ICS, inhaled corticosteroids; LABA, long-acting beta-agonist; LAMA, long-acting muscarinic antagonist; pMDI, pressurised metered-dose inhaler; SABA, short-acting beta-agonist; SAMA, short-acting muscarinic antagonist; SMI, soft mist inhaler.

Based on inhaler device type, the DPI was the most studied inhaler category (23 studies), followed by the pMDI (15 studies), with only two studies including SMIs in the analysis. The ICS/LABA DPI was the most studied inhaler drug category (13 studies), followed by ICS/LABA/LAMA (5 studies) and SABA (5 studies) (figure 3).

In total, 42 studies reported one outcome, 19 studies reported two outcomes and 2 studies reported four outcomes (online supplemental file 4) across the PPP framework. The studies were further classified based on the primary and secondary outcomes under the PPP framework (online supplemental file 4). Based on this classification, 23 studies had primary outcomes focused on people—19 with outcomes on patients and 4 with outcomes on healthcare professionals. For the process aspect, 12 studies had primary outcomes on the process of change in inhaler sustainability, while 38 studies reported primary outcomes on sustainability from the product perspective.

**Figure 4:**
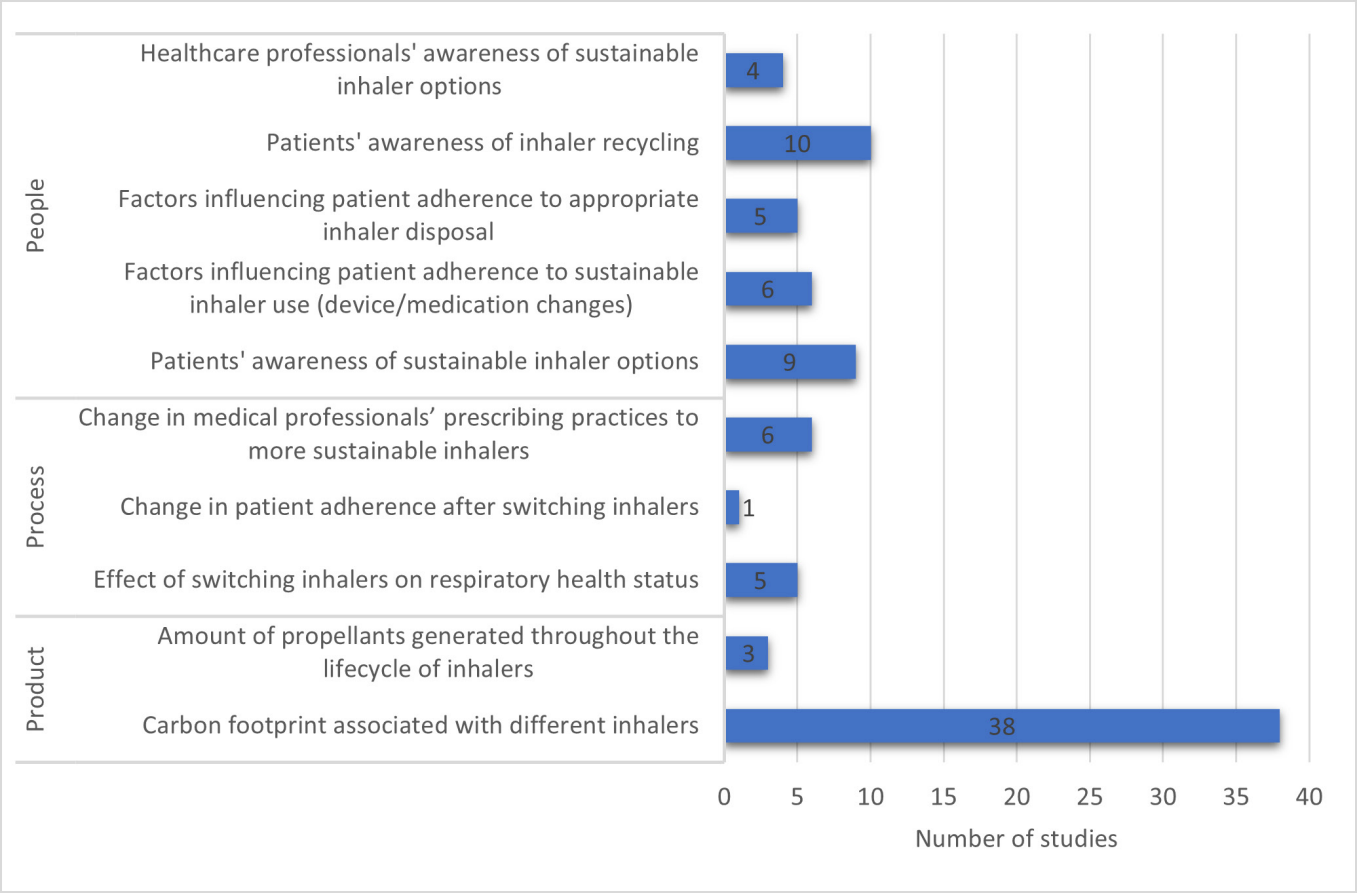
Sustainability outcome themes.

In total, based on both primary and secondary outcomes from all included studies, the carbon footprint of inhalers was the most common study focus, with a total of 38 studies (figure 4). This is followed by patient awareness of inhaler recycling (10 studies), and awareness of both inhaler recycling and sustainable inhaler options among patients (9 studies). An overview of the study’s classification based on combined primary and secondary outcomes of included studies within the tripartite framework is shown in figure 4.

### People outcome

#### Patient perspective

A total of thirty studies focused on the people outcome from the patient’s perspective (figure 4).

##### Patients’ awareness of sustainable inhaler options

Nine studies^2831 34^,^37 41 43 63^ focused on patients’ awareness, preferences and behaviours regarding the environmental impact of inhalers. Across the studies, there was a recurring theme of limited patient awareness regarding the carbon footprint of inhalers, with baseline awareness rates ranging from 21% to 70%.^28 31 41 63^ However, in some studies,^28 31 41 63^ patients expressed a willingness to switch to more sustainable options if effectiveness was maintained, with support from prescribers or pharmacists often cited as a key factor in decision-making.

Across the studies reviewed, recurring themes influencing patients to switch to sustainable inhalers included perceived effectiveness, ease of use and better patient education on sustainability. Barriers to switching devices include concerns about device usability, efficacy and cost, particularly in countries where out-of-pocket expenses vary by healthcare system. These findings emphasise the need for context-specific strategies to support sustainable inhaler use.

##### Factors influencing patient adherence to sustainable inhaler use (device/medication changes)

Six studies^3141 58 64^,^66^ focused on inhaler use and patient preferences as factors that influence adherence to inhaler use. Across the studies, key factors include ease of use, portability and sustainability considerations. Rothwell *et al*^41^ showed that patients valued the ease of use (92%) and portability (68%) of inhalers, with 69% expressing concern about the carbon footprint. Similarly, Baggott *et al*^64^ found that patients preferred small, portable inhalers with dose counters. In line with these findings, Savage *et al*^31^ found that 79% of patients using different device types for maintenance and reliever therapy demonstrated good adherence, compared with 65% of those using consistent devices, indicating that adherence may be influenced by factors beyond device consistency.

Beliefs about asthma and the role of medications further influenced adherence, as seen in the study by Rojano *et al*.^66^ Patients who believed they had asthma all the time or understood the importance of medication use, even when symptom-free, were more likely to adhere to treatment. Conversely, those who only valued short-acting medications were less likely to adhere. These studies collectively highlight the complex interplay of device preferences, beliefs, patient education and awareness of environmental sustainability in inhaler use.

##### Patients’ awareness of appropriate inhaler disposal and recycling

10 studies^12 28 30 35 37 41 43 44 67 68^ focused on patient awareness and behaviours related to inhaler disposal and recycling, which represent key aspects of the environmental impact of inhalers. A common finding across studies is the lack of awareness regarding proper disposal methods. Various studies examined the awareness and practices related to inhaler recycling and disposal, showing significant gaps in patient knowledge and engagement. Rothwell *et al*^41^ found that 78% of patients were unaware that inhalers could not be recycled through local council programmes. Similarly, Blyth *et al*^43^ reported that 80% of patients did not know that inhalers should be recycled at pharmacies, with all participants disposing of inhalers in household waste. Similarly, Fullwood *et al*^67^ found that 83% of patients disposed of their inhalers in general waste, and Florman *et al*^37^ reported that only 10.3% of patients returned used inhalers to a pharmacy for correct disposal. Improper disposal is a significant environmental concern, as it results in the release of residual propellant gases that contribute to greenhouse gas emissions.

Studies by Murphy *et al*^44^ and Nurse *et al*^30^ indicated that more patient education could significantly improve recycling rates, with 77% of participants in Murphy *et al*’s study willing to recycle via postal methods and 80% of respondents expressing willingness to recycle if they had better information. Nurse *et al*’s^30^ findings show a marked improvement in awareness of correct inhaler disposal options after an educational intervention via posters, from 0% to 50% after two rounds of intervention.

Khan *et al*^12^ analysed disposal recommendations from manufacturers in Canada and showed that a significant proportion (60.5%) advised patients to ‘discard inhalers’ without specifying where to dispose of the inhalers. Only 4.7% of product information leaflets mention returning used inhalers to the pharmacy, reflecting a lack of standardised guidance. Fullwood *et al*^67^ and d’Ancona *et al*^35^ indicated similar concerns, with many patients unaware of when inhalers were empty, what the appropriate disposal methods were, and what recycling options were available.

##### Factors influencing patient adherence to appropriate inhaler disposal

Five studies^29 30 41 44 63^ focused on the factors affecting the disposal practices of patients using inhalers. Across studies, there is a strong focus on improving inhaler disposal, recycling and patient awareness. Various strategies, from pharmacy involvement to postal recycling, were evaluated, with all showing potential for reducing inhaler waste. Liatsikos *et al*^29^ identified that while 64% of pharmacies accepted inhalers for safe disposal, only 28% offered recycling, with sustainability and financial incentives being key motivators for service provision. Murphy *et al*^44^ assessed the feasibility of a postal recycling scheme, reporting high patient satisfaction (90%) and significant participation driven by environmental concerns (94%). Most inhalers returned were pMDIs (77%), indicating that such schemes could have a substantial environmental impact. Nurse *et al*^30^ explored the impact of awareness campaigns in paediatric asthma clinics, finding that nearly all patients and staff (97%) rated sustainability as highly important.

### Healthcare professional perspective

#### Healthcare professionals’ awareness of sustainable inhaler options

Four studies^15 19 40 43^ investigated healthcare professionals’ knowledge and practices regarding inhaler prescribing and environmental sustainability. Walpole *et al*^40^ found that only 46.1% of prescribers in the UK felt confident discussing lower-carbon inhaler options, and 55.9% were unaware that inhalers could be recycled at pharmacies. Blyth *et al*^43^ similarly reported that 66% of staff did not know about inhaler recycling, and 50% were unaware of the varying carbon footprints of different inhalers.

A study by Bosnic-Anticevich *et al*^15^ highlighted that healthcare professionals in the UK (51%) were more likely to consider the climate impact of inhalers than those in the Asia-Pacific (41%) and Australia (17%). However, the environmental impact was the least considered factor when switching inhalers. Stanley *et al*^19^ found that while 50% of team members correctly identified the carbon footprint of pMDIs, only one had previously discussed greener alternatives with patients.

### Process outcome

#### Effect of switching inhalers on respiratory health status

Five studies^13 17 26 39 45^ focused on the effect of switching inhalers on respiratory health. Most of the studies focused on switching inhalers from pMDIs to DPIs and their clinical and environmental impacts.^17 26 39 45^ The study by Janson *et al*^26^ demonstrated significant improvements in asthma control and lung function after patients with asthma, COPD and asthma-COPD overlap switched from pMDI to DPI. Among patients with asthma, Asthma Control Test (ACT) scores increased by 58%, and forced expiratory volume in 1 s (FEV₁%) improved by 13.6%. Patients with COPD experienced a 28.5% reduction in COPD Assessment Test (CAT) scores and a 14.6% increase in FEV₁. Similarly, Yiu *et al*^39^ showed that patient education sessions on device use led to improved asthma control, with 96% of patients opting to continue on DPIs after 3 months and 3% increase in ACT scores.

Woodcock *et al*^45^ found that patients switching to fluticasone/vilanterol DPI showed better asthma control (76% responders vs 63% on usual care) and used about one fewer salbutamol inhaler over 12 months compared with those who stayed on pMDI. Nagel *et al*^13^ compared lung delivery efficiency and carbon emissions for two inhalers, showing that adding a spacer increased lung deposition without increasing emissions. Gálffy *et al*^17^ showed that switching to the Easyhaler DPI in real-life settings resulted in substantial improvements in asthma control from 13% to 78% (ACT≥20), and better COPD control rose from 24% to 68% (CAT≤20). There was also a significant increase in quality of life scores (p<0.001) and increased patient satisfaction, with 74% rating Easyhaler as ‘very good’ compared with 13% for pMDI.

#### Change in patient adherence after switching inhalers

One study, Alvarez-Gutiérrez *et al*^58^ focused on patient adherence after switching inhalers and also evaluated patient preferences, satisfaction and ease of training when switching from other DPIs to the Easyhaler DPI. Preswitch adherence was 45.5%, increasing marginally to 47.5% postswitch. Satisfaction was significantly higher with the Easyhaler (The Feeling of Satisfaction with Inhaler (FSI-10): 31.8 vs 29.1; p<0.001), with 38.4% of patients exclusively preferring the Easyhaler compared with 15.3% who preferred their prior devices. While the study’s design allows for a snapshot of patient preference, longitudinal data would be beneficial for understanding long-term adherence, satisfaction and measures to support behaviour change for sustained adherence.

#### Change in medical professionals’ prescribing practices towards more sustainable inhalers

Six studies^19 32 43 46 53 69^ focused on inhaler prescribing practices, their environmental impact and potential areas for improvement. Stanley *et al*^19^ conducted a chart audit and survey, which showed that 29% of patients initially prescribed pMDIs were switched to DPIs or SMIs, and 40% of newly diagnosed patients were started directly on these alternatives. However, staff knowledge and confidence regarding the inhaler’s carbon impact were limited. Similarly, Blyth *et al*^43^ examined prescribing practices and found that 50% of patients had the potential to switch to more sustainable inhalers, highlighting an opportunity for improving inhaler sustainability.

Two studies examined national prescribing patterns and associated emissions. Nagasaki *et al*^69^ reported that in Japan, 22.6% of inhalers prescribed in 2019 were pMDIs, which contributed disproportionately to the sector’s carbon footprint compared with the 77.4% prescribed as DPIs or SMIs. Similarly, Janson *et al*^53^ compared the carbon footprint of inhalers in England and Sweden in 2017 and found that 70% of inhalers in England were pMDIs, compared with 13% in Sweden, reflecting more environmentally sustainable inhaler prescription and use in Sweden.

Two other studies focused on medication type. Wilkinson *et al*^32^ assessed SABA use across five European countries. The UK had the highest SABA use at 70.2%, contributing to higher greenhouse gas emissions compared with other countries. Crooks *et al*^46^ evaluated the impact of national incentives on SABA prescribing in England and found no significant reduction in total SABA prescriptions despite the introduction of Investment and Impact Fund incentives, suggesting the need for stronger measures to reduce SABA overuse.

### Product outcome

#### Carbon footprint associated with different inhalers

We found 38 studies^15^,^7 13 14 16 18 20^ examining the carbon footprint of inhalers across various stages of the product life cycle, including raw materials, manufacturing, distribution, use, disposal, recycling and total emissions. 12 studies focused specifically on inhaler use patterns, while the remaining studies addressed a combination of medication content and device type in their assessment of carbon footprint. Reporting methods varied, with carbon footprints expressed per actuation, medication dose, device or year, depending on each study’s objectives. Five inhaler medication classes are noteworthy.

##### Short-acting beta agonists

Five studies,^42 49 53 61 70^ including Sosnowski *et al*^70^ and Jeswani and Azapagic,^42^ showed that the total life cycle carbon footprint of SABA inhalers like fenoterol and salbutamol ranges from 17 to 23.5 kg CO_2_e per inhaler, while Alzaabi *et al*^6^ reported total SABA emissions across 28 countries between 2019 and 2020 at 2.7×10⁹ kg CO_₂_e. Per capita SABA use and emissions were highest in Australia, the Middle East and high-income countries, with over 90% of SABA prescriptions going to patients already overusing them, highlighting a clear link between poor asthma control and environmental impact.

##### Inhaled corticosteroids

Sosnowski *et al*^70^ estimated that the life cycle greenhouse gas emissions of individual ICS inhalers, such as ciclesonide and fluticasone, are approximately 12–15 kg CO_₂_e per device, which is environmentally significant. Inget *et al*^59^ estimated annual per-patient life cycle greenhouse gas emissions for ICS inhalers, such as budesonide and beclometasone, at approximately 577–650 kg CO_₂_e. Panigone *et al*^71^ also compared different propellants with the same medication content, showing that switching from hydrofluoroalkane (HFA)-134a to HFA-152a can reduce emissions drastically, from 0.0831 kg CO_₂_e/actuation to 0.00939 kg CO_₂_e/actuation for a 100 μg/actuation inhaler. These findings show that while ICS inhalers contribute substantially to inhaler-related emissions from frequent use, switching to lower-GWP propellants and non-propellant devices can significantly reduce their footprint.

##### Long-acting beta agonists

Inget *et al*^59^ reported a reduction in estimated annual per-patient life cycle emissions for formoterol, from 573.69 kg CO_2_e (in 2019) to 511.32 kg CO_2_e (in 2023), likely linked to the calculation method without any changes to the product itself.

##### Combination inhalers (ICS/LABA)

The contribution of combination ICS/LABA inhalers to the carbon footprint varies by medication and device type. Sosnowski *et al*^70^ showed that a single salmeterol/fluticasone inhaler produces about 14.5 kg CO_₂_e over its life cycle, while Inget *et al*^59^ estimated that annual per-patient emissions for budesonide-formoterol fell from 514 kg CO_₂_e in 2019 to 452 kg CO_₂_e in 2023, while salmeterol-fluticasone declined from 602 kg CO_₂_e to 540 kg CO_₂_e. Jeswani and Azapagic,^42^ as well as Janson *et al,*^53^ independently found that combination inhalers like Seretide Accuhaler, a DPI, produce 0.06 kg CO_₂_e per device or approximately 0.9–11 kg CO_₂_e per year, while the Seretide Evohaler, a pMDI, can generate up to 234 kg CO_₂_e per year. The shift to DPIs significantly reduces the environmental impact.

##### Long-acting muscarinic antagonists

Hansel *et al*^62^ measured carbon footprint across the inhaler life cycle, showing that LAMA SMIs emit around 4–4.5 kg CO_₂_e per device, compared with 14.6 kg CO_₂_e per device for a pMDI. The higher carbon footprint of pMDIs compared with SMIs for LAMA is primarily driven by propellant emissions during use and disposal.

### Amount of propellants generated throughout the life cycle of inhalers

Three studies^7 42 70^ reported on the environmental impact of inhaler propellants, particularly hydrofluorocarbons (HFCs), which contribute significantly to the carbon footprint of pMDIs. Sosnowski *et al*^70^ showed that propellant emissions account for 96–98% of the total carbon footprint, with HFA-134a generating 65–97.5 kg CO_2_e per single dose. Jeswani and Azapagic^42^ also showed that 3.6 g of HFA-134a emissions were released during the inhaler’s end-of-life.

In the study by Jeswani and Azapagic,^7^ various HFCs, including the newer HFC-152a, were assessed for environmental impact. The study found that HFC-134a had the highest environmental release of propellants (166.7 mg/dose) compared with HFC-227ea (162.7 mg/dose), and HFC-152a had the least (110 mg/dose). Collectively, these findings indicate that propellant emissions during use and disposal are the main contributors to the high carbon footprint of pMDIs, underscoring the value of transitioning to propellant-free devices, such as DPIs or SMIs, when clinically appropriate.

## Discussion

To our knowledge, this study represents the first systematic review of inhaler sustainability in asthma and COPD care. This review highlights several critical insights regarding the inter-relationship between inhaler design, use, prescription, disposal and the environmental impact of inhaler choices, from which we suggest evidence-based actionable recommendations (table 1). The studies reviewed cover various themes, including patient awareness, disposal practices, adherence to sustainable inhaler use and the healthcare professionals’ role in promoting environmentally friendly options. Collectively, these findings point towards a need for increased awareness, education and action to improve inhaler sustainability without compromising patient outcomes.

**Table 1:** Recommendations based on the outcomes of the systematic review

PPP domain	Recommendations
People	Integrate a short ‘green inhaler’ discussion at each asthma/COPD review using a patient decision aid. GPs, respiratory physicians and prescribing nurses should flag SABA overuse and review those patients. Patients should have an inhaler technique check at first prescription, yearly reviews and at the beginning and 6 weeks after any inhaler device switch. Patients should be educated by prescribers and pharmacists on returning inhalers to pharmacies.
Process	Embed a ‘low carbon’ prescribing module in undergraduate medical, pharmacy and nursing programmes. Require completion of an annual refresher course on the ‘low carbon’ prescribing module with physicians, GPs, nurses and pharmacists. List DPIs and SMIs as first-line choices in the medicines formulary, with pMDIs allowed only when clinically justified. Update e-prescribing systems to default to the lowest-carbon device that meets the prescription, display the carbon footprint difference if a pMDI is selected and log prescriber overrides for audit and feedback.
Product	Make DPI and SMI devices the first-line option in local formularies when clinically appropriate, using the NICE or similar guidelines and cost-effectiveness data (including environmental costs) to justify exceptions for pMDIs. Require all inhaler manufacturers to include a disposal statement and/or a pharmacy-return icon on every pack and patient information leaflet. Embed inhaler returns into the Community Pharmacy Contractual Framework, with monthly reporting of returned inhaler numbers, which can be tied to sustainability points for the pharmacies. Fund pilot schemes to trial other inhaler return options, such as drop boxes in GP surgeries or pharmacies, measuring uptake, cost and carbon savings against pharmacy returns. Amend UK EPR regulations so that manufacturers of high-GWP pMDIs finance end-of-life collection and recycling, thereby creating a financial incentive to shift towards low-GWP devices.

COPD, Chronic Obstructive Pulmonary Disease; DPI, dry powder inhaler; EPR, Extended Producer Responsibility; GP, General Practitioner; GWP, global warming potential; NICE, National Institute for Health and Care Excellence; pMDI, pressurised metered-dose inhaler; PPP, People-Process-Product; SABA, short-acting beta-agonist; SMI, soft mist inhaler.

From the patient’s perspective, the findings suggest a significant gap in awareness regarding the environmental impact of inhalers. While there is a willingness among patients to switch to more sustainable options, provided they are effective and convenient, many remain unaware of the carbon footprint of their inhalers. This lack of awareness extends to proper disposal and recycling. Three studies showed that the vast majority of patients disposed of their inhalers through household waste^37 43 67^ and were unaware that pharmacies offer disposal or recycling options. Of note, patients expressed a willingness to engage in more sustainable practices, such as using postal recycling schemes or returning inhalers to pharmacies, when adequately informed. These findings indicate an opportunity for interventions aimed at raising patient awareness, improving access to disposal/recycling options and aligning patient preferences with more sustainable inhaler choices.

Regarding adherence to sustainable inhaler use, the studies identified key factors influencing patients’ willingness to switch inhaler devices. Ease of use, portability and a consistent inhaler type were significant predictors of adherence.^3141 58 64^,^66^ Sustainability was an important consideration for many patients, but concerns about cost, convenience and efficacy often created barriers to switching. The evidence suggests that addressing these concerns through patient education, prescriber support and accessible, affordable and sustainable alternatives could significantly improve adherence to environmentally sustainable inhalers. Moreover, addressing patient beliefs about asthma management, particularly regarding long-term medication use even in the absence of symptoms, is crucial to improving overall adherence rates.

The studies examining inhaler disposal and recycling practices underscore the need for better education and clearer guidance on inhaler disposal. Patients remain unaware of proper disposal methods, and the studies revealed a widespread practice of disposing of inhalers in household waste. Pharmacy and postal recycling schemes have the potential to significantly reduce this waste, provided patients are made aware of and have access to such options. Healthcare professionals have a role in educating patients about these options. There is also a need for creating a national-level recycling programme, which could further improve appropriate inhaler disposal.

From the healthcare professional’s perspective, a notable gap exists in the awareness and confidence of prescribers when discussing sustainable inhaler options. Most healthcare professionals lack knowledge of the environmental impact of inhalers and appropriate inhaler disposal methods. The studies suggest that incorporating sustainability into clinical training and subsequently into prescribing practices is essential. This would not only promote the use of lower-carbon inhalers, such as DPIs, but also increase patient confidence in switching to these alternatives, thereby supporting both patient and environmental health.

The process perspective highlights the clinical and environmental benefits of switching from pMDIs to DPIs. Five studies^13 17 26 39 45^ consistently showed that patients who switched to DPIs experienced improved asthma control, better adherence and reduced rescue inhaler use, all the while lowering the carbon footprint of their treatment. These findings suggest that transitioning patients from pMDIs to DPIs could have the dual benefit of improving health outcomes while contributing to environmental sustainability. However, more long-term data are needed to fully assess the impact of these transitions on adherence, disease control and patient satisfaction with use.

The product perspective focused on the carbon footprint of different inhaler types, showing that DPIs and SMIs have a significantly lower environmental impact than pMDIs, which rely on HFC propellants. HFC emissions from pMDIs account for most of their carbon footprint, and studies demonstrate that transitioning to alternative propellants or switching to DPIs and SMIs could drastically reduce emissions. While current guidance supports switching from pMDIs to DPIs due to their lower carbon footprint,^4 72^ it is unclear if the development of low-GWP propellants (eg, HFA-152a) may change this recommendation as they become commercially available.

The 2024 revision of the European Union Fluorinated greenhouse gases (EU F-gas) Regulation^73^ mandates a reduction in the use of high-GWP HFC propellants by 2030, which will significantly limit the availability of conventional pMDIs. This regulatory shift is likely to accelerate the adoption of DPIs, SMIs and low-GWP pMDIs, such as those using HFA-152a. As DPIs and SMIs contain no propellant, they will likely remain the least environmentally impactful option, unless future evidence demonstrates that low-GWP propellant inhalers can match or surpass them in terms of total emissions. Future environmental comparisons will need to account not only for propellant emissions but also for patient adherence to inhaler dose and correct inhaler use, both of which significantly influence treatment effectiveness and overall environmental impact.

The main limitation of this study is the wide heterogeneity of study types and content included, making it difficult to meta-analyse the data. Under these conditions, a forest plot would risk oversimplifying findings that are highly context-dependent and methodologically diverse, potentially misrepresenting the comparability of results. In addition, 46% of the studies were small studies published in conference abstracts with limited data reporting, which limits the robustness of the data reported. Data were also limited to English language papers, limiting the data available for this review.

## Conclusions

The review demonstrates a clear opportunity to align clinical and environmental goals within the asthma and COPD care pathway. We chose to propose a framework and a set of actionable recommendations for intervention, including improving patient awareness of sustainable inhaler options and disposal practices, supporting healthcare professional training on the environmental impact of inhalers, and facilitating the switch from pMDIs to DPIs or other low-emission alternatives. By addressing these gaps, we can promote the dual goals of improving respiratory health outcomes and reducing the environmental footprint of inhalers. Future research might address each of our proposed actions in turn or group actions under the PPP framework to prioritise them and enable better future evidence synthesis.

## Supplementary material

10.1136/bmjopen-2024-098052 online supplemental file 1

10.1136/bmjopen-2024-098052 online supplemental file 2

10.1136/bmjopen-2024-098052 online supplemental file 3

10.1136/bmjopen-2024-098052 online supplemental file 4

## Data Availability

All data relevant to the study are included in the article or uploaded as supplementary information.
