# Supplementary material for: Inhaler sustainability in asthma and COPD care: a systematic review
Source: BMJ Open. 2025 Jul 25;15(7):e098052. doi: 10.1136/bmjopen-2024-098052 (PMC12306229; doi:10.1136/bmjopen-2024-098052)
Supplement: online supplemental file 1 [file bmjopen-15-7-s001.docx]

## Supplementary file 1: Search strategy

1. Ovid Medline
   Filters: Language (English), Year (2014-2024)

| **Population** | **Intervention** | **outcomes** |
| --- | --- | --- |
| Asthma. ab,ti  COPD. ab,ti | - inhaler*.ab,ti. - inhalation device. ab,ti. - Inhaler design. ab,ti - inhaler material*. ab,ti - Inhaler component*. ab,ti - Inhaler part*. ab,ti - Inhaler device*. ab,ti - metered dose inhaler*. ab,ti - dry powder inhaler*. ab,ti - nebulizer*. ab,ti - soft mist inhaler*. ab,ti - inhaler use. ab,ti | - recycl*.ab,ti - disposal practice*.ab,ti - disposal.ab,ti - recycling practice*.ab,ti - recycling initiative*.ab,ti - recycling program*.ab,ti - recycled material*.ab,ti - waste management.ab,ti - waste disposal.ab,ti - collection scheme*.ab,ti - disposal practices.ab,ti - reuse.ab,ti - recover*.ab,ti - waste reduction strateg*.ab,ti - Medical plastics recycl*.ab,ti - return program*.ab,ti - Sustainable design*.ab,ti - Sustainab*.ab,ti - eco-friendly.ab,ti - environmentally friendly.ab,ti - green.ab,ti - environmental impact.ab,ti - carbon footprint.ab,ti - environmental sustainability.ab,ti - environmental assessment.ab,ti - environmental evaluation.ab,ti - environmental analysis.ab,ti - polic*.ab,ti - regulatory framework.ab,ti - government regulation*.ab,ti - legislation.ab,ti - policy guideline*.ab,ti |

1. **HTA Database (simple search)**

‘Inhaler’ (Free term) no limits

1. **Scopus**

'inhaler' AND 'recycling OR sustainability' (search: Title, Abstracts, keywords)

Filters: Language (English), Year (2014-2024)

1. **Cochrane Library**
   ‘inhaler and recycling’ (search: Title, Abstracts, keywords)
2. **Basefield Search Engine**

‘inhaler and recycling’ (All fields)

1. **Google Scholar**

'asthma or COPD' and 'inhaler' and 'recycle or recycling or sustainability'

Limits (First 20 pages)

1. **Refseek**

'asthma or COPD' and 'inhaler' and 'sustainability or recycling' year:[2014 TO 2024]
