# Supplementary material for: Inhaler sustainability in asthma and COPD care: a systematic review
Source: BMJ Open. 2025 Jul 25;15(7):e098052. doi: 10.1136/bmjopen-2024-098052 (PMC12306229; doi:10.1136/bmjopen-2024-098052)
Supplement: online supplemental file 2 [file bmjopen-15-7-s002.docx]

## Supplementary file 2: Inclusion/ Exclusion criteria

| **Domain** | **Inclusion criteria** | **Exclusion criteria** |
| --- | --- | --- |
| Population | - Patients with respiratory conditions requiring inhaler use - Healthcare professionals involved in prescribing, teaching, and managing inhaler use | Other population |
| Exposure | Exposure to any aspect of inhaler sustainability, including awareness, perceptions, behaviours, or practices related to inhaler use, device type, environmental impact, prescribing, disposal, and recycling. | Studies that don’t address sustainability |
| Outcomes | 1. Effect of switching inhalers on respiratory health  o Measured by changes in exacerbation rates and overall respiratory health status.  2. Patients' awareness of sustainable inhaler options  o Assessed by surveys or questionnaires assessing awareness  3. Change in patient adherence after switching inhaler regimens  o Measured by adherence rates from prescription refill data, electronic medication monitoring data, or self-report tools.  4. Factors influencing patient adherence to the use of sustainable inhaler regimens  o Identified through qualitative interviews and or surveys  5. Factors influencing patient adherence to appropriate inhaler disposal  o Identified through surveys, qualitative interviews, and observational studies.  6. Patients’ awareness of inhaler recycling  o Measured by surveys or questionnaires assessing awareness  7. Healthcare professionals' awareness of sustainable inhaler options  o Measured by surveys or questionnaires assessing awareness before and after targeted educational interventions/ policy changes.  8. Change in medical professionals’ prescribing practices to more sustainable inhalers  o Measured by prescription audit data and surveys of prescribing behaviour.  9. Carbon footprint associated with different inhalers, measured in CO2 equivalents per kilogram  o Calculated based on inhaler manufacturing, use, and disposal lifecycle analysis.  10 Amount of plastics generated throughout the lifecycle of inhalers  o Measured in kilograms of plastic waste produced from manufacturing to disposal.  11 Amount of propellants generated throughout the lifecycle of inhalers  o Measured in kilograms or litres of propellants used/or released during the lifecycle of the inhalers. | Studies that do not report at least one of the outcomes of interest as noted under the inclusion criteria.  Non-human models, animal studies, |
| Study Design | 1. All primary studies  2. Secondary studies on lifecycle analysis | Systematic review, literature reviews, |
| Time period | January 1, 2014 to April 30, 2024 | Studies published before 2014 or after April 30, 202 |
| Language | English language | Studies published in languages other than English |
