# Supplementary material for: Inhaler sustainability in asthma and COPD care: a systematic review
Source: BMJ Open. 2025 Jul 25;15(7):e098052. doi: 10.1136/bmjopen-2024-098052 (PMC12306229; doi:10.1136/bmjopen-2024-098052)
Supplement: online supplemental file 3 [file bmjopen-15-7-s003.docx]

## Supplementary file 3: Quality Assessment

| Study ID | Source of Publication | Was there a clear statement of the aims of the research? | Is the methodology appropriate? | Was the research design appropriate to address the aims of the research? | Was the recruitment strategy appropriate to the aims of the research? | Is there a clear statement of findings? | Was the data analysis sufficiently rigorous? | Was the outcome data reportedly comprehensively | Was the outcome data reported completely? |
| --- | --- | --- | --- | --- | --- | --- | --- | --- | --- |
| Herrera 2024 | Conference | Yes | Yes | Yes | Unsure | Yes | Yes | Yes | No |
| Goulet 2017 | Journal | Yes | Yes | Yes | Yes | Yes | Unsure | No | Yes |
| Wilkinson 2019 | Journal | Yes | Yes | Yes | Yes | Yes | Unsure | Unsure | No |
| Walpole 2021 | Journal | Yes | Yes | Yes | Unsure | Yes | Yes | Yes | Yes |
| Sosnowski 2023 | Journal | Yes | Yes | Yes | Yes | Yes | Unsure | Yes | No |
| Rothwell 2024 | Journal | Yes | Yes | Yes | Unsure | Yes | Yes | Yes | Yes |
| Nagasaki 2023 | Journal | Yes | Yes | Yes | Yes | Yes | Yes | Yes | Yes |
| Jeswani 2020 | Journal | Yes | Yes | Yes | Yes | Yes | Yes | Yes | Yes |
| HÃ¤nsel 2019 | Journal | Yes | Yes | Yes | Yes | Yes | Unsure | Yes | Yes |
| Fulford 2021 | Journal | Yes | Unsure | Yes | Yes | Yes | Yes | Unsure | Unsure |
| Baggott 2020 | Journal | Yes | Yes | Yes | Yes | Yes | Yes | Yes | Yes |
| Liatsikos 2021 | Conference | Yes | Yes | Yes | Unsure | Yes | Yes | Yes | Yes |
| OrtsÃ¤ter 2020 | Journal | Yes | Yes | Yes | Yes | Yes | Unsure | Yes | Yes |
| Inget 2023 | Journal | Yes | Yes | Yes | Yes | Yes | Yes | No | Yes |
| Jeswani 2019 | Journal | Yes | Yes | Yes | Yes | Yes | Yes | Yes | Yes |
| Quantz 2023 | Journal | Yes | Yes | Yes | Yes | Yes | Yes | Yes | Yes |
| Blyth 2023 | Journal | Yes | Yes | Yes | Unsure | Yes | Unsure | No | No |
| OrtsÃ¤ter 2019 | Journal | Yes | Yes | Yes | Yes | Yes | No | Yes | No |
| Murphy 2023 | Journal | Yes | Yes | Yes | Yes | Yes | Yes | Yes | Yes |
| Nurse 2022 | Conference | Yes | Yes | Yes | Yes | Yes | Unsure | Unsure | Unsure |
| Savage 2023 | Conference | Yes | Yes | Yes | Unsure | Yes | Yes | Yes | Yes |
| Alvarez-Gutierrez 2021 | Journal | Yes | Yes | Yes | Unsure | Yes | Yes | Yes | Yes |
| Valero 2017 | Conference | Yes | Yes | Yes | Unsure | Yes | No | Yes | Yes |
| Galffy 2019 | Conference | Yes | No | Yes | Unsure | Yes | Yes | Yes | Yes |
| Rojano 2019 | Journal | Yes | Yes | Yes | Yes | Yes | Yes | Yes | Yes |
| Varghese 2020 | Conference | Yes | Yes | Yes | Yes | Yes | Unsure | Unsure | No |
| Janson 2020 | Journal | Yes | Yes | Yes | Yes | Yes | Yes | Yes | Yes |
| Panigone 2020 | Journal | Yes | Yes | Yes | Yes | Yes | Unsure | Yes | No |
| Wilkinson 2021 | Conference | Yes | Yes | Yes | Yes | Yes | Unsure | Yes | Yes |
| Borenius 2021 | Conference | Yes | Yes | Yes | Unsure | Yes | Unsure | No | No |
| Hunt 2021 | Conference | Yes | Unsure | Unsure | Unsure | Yes | No | No | Unsure |
| Khan 2021 | Conference | Yes | Yes | Yes | Yes | Yes | No | Yes | Yes |
| Beeh 2021 | Conference | Yes | Yes | Yes | Yes | Yes | Unsure | No | No |
| Aumonier 2021 | Conference | Yes | Unsure | Yes | Unsure | Yes | Unsure | No | No |
| Janson 2021 | Conference | Yes | Yes | Yes | Yes | Yes | Yes | Yes |  |
| D'Ancona 2021 | Conference | Yes | Yes | Yes | Unsure | Yes | Yes | Yes | Yes |
| Vartiainen 2021 | Conference | Yes | Yes | Yes | Yes | Yes | Unsure | No | Unsure |
| Beeh 2021 | Conference | Yes | Yes | Unsure | No | Unsure | Unsure | Unsure | Unsure |
| Pernigotti 2021 | Journal | Yes | Yes | Yes | Yes | Yes | Unsure | Yes | Yes |
| Choubey 2022 | Conference | Yes | Yes | Yes | No | No | Unsure | No | No |
| Aumonier 2022 | Conference | Yes | No | No | No | Yes | Unsure | Unsure | No |
| Kponee-Shovein 2022 | Journal | Yes | Yes | Yes | Yes | Yes | Yes | Yes | Yes |
| Kponee-Shovein 2022 | Journal | Yes | Yes | Yes | Yes | Yes | Unsure | Yes | Yes |
| TenHave 2022 | Journal | Yes | Yes | Yes | Yes | Yes | Unsure | Yes | Yes |
| Fullwood 2022 | Journal | Yes | Yes | Yes | Unsure | Yes | Yes | Yes | Yes |
| Janson 2022 | Journal | Yes | Yes | Yes | Yes | Yes | Unsure | Unsure | Unsure |
| D'Ancona 2022 | Conference | Yes | Yes | Yes | Yes | Yes | Yes | Yes | Yes |
| Bell 2022 | Conference | Yes | Yes | Yes | Yes | Yes | Unsure | No | No |
| Bosnic-Anticevich 2023 | Conference | Yes | Yes | Yes | Unsure | Yes | Unsure | No | No |
| Narendran 2023 | Journal | Yes | Yes | Yes | Yes | Yes | Yes | Yes | Yes |
| Janson 2023 | Journal | Yes | Yes | Yes | Yes | Yes | Unsure | Yes | Yes |
| Nagel 2023 | Conference | Yes | Yes | Yes | Yes | Yes | Unsure | Yes | Yes |
| Woodcock 2022 | Journal | Yes | Yes | Yes | Yes | Yes | Unsure | Yes | Yes |
| Gagne 2023 | Journal | Yes | Yes | Yes | Yes | Yes | Yes | Yes | Yes |
| Alzaabi 2023 | Journal | Yes | Yes | Yes | Unsure | Yes | Yes | Yes | Yes |
| Wilkinson 2022 | Conference | Yes | Yes | Yes | Unsure | Yes | Yes | No | Yes |
| Florman 2022 | Conference | Yes | Yes | Yes | Unsure | Yes | Yes | Unsure | Unsure |
| Stanley 2023 | Conference | Yes | Yes | Yes | Unsure | Yes | Unsure | Unsure | Unsure |
| Haughney 2023 | Conference | Yes | Yes | Yes | Yes | Yes | Yes | Yes | Yes |
| Janson 2023 | Conference | Yes | Yes | No | Unsure | Yes | Unsure | No | Unsure |
| Yiu 2023 | Conference | Yes | Yes | Yes | Unsure | Yes | Yes | No | No |
| Hatter 2024 | Journal | Yes | Yes | Yes | Unsure | Yes | Yes | No | Unsure |
| Crooks 2024 | Journal | Yes | Yes | Yes | Unsure | Yes | Yes | Yes | Yes |
