# Supplementary material for: Inhaler sustainability in asthma and COPD care: a systematic review
Source: BMJ Open. 2025 Jul 25;15(7):e098052. doi: 10.1136/bmjopen-2024-098052 (PMC12306229; doi:10.1136/bmjopen-2024-098052)
Supplement: online supplemental file 4 [file bmjopen-15-7-s004.docx]

Supplementary file 4: Overview of studies

| **Study ID** | **Aim of study** | **Key message** | **Secondary message category** | **Key secondary message** |
| --- | --- | --- | --- | --- |
| **People: patient n=19 studies (primary)** | | | | |
| Alvarez-Gutierrez 2021 | Primary objective: assess preference and satisfaction with the Easyhaler in a population of patients previously treated with a variety of DPIs. Secondary objectives: compare the ease of training in the use of Easyalher with that of other DPIs and to estimate patient satisfaction and ease-of-use of inhaler devices. | Factors influencing patient adherence to sustainable inhaler use (device/medication changes) | Process | Change in patient adherence after switching inhalers |
| Baggott 2020 | To explore adult patients' experiences of asthma and preferences for asthma management. | Factors influencing patient adherence to sustainable inhaler use (device/medication changes) | - | - |
| Blyth 2023 | To reduce the carbon footprint of inhaler prescribing in the hospital by exploring current prescribing practices, identifying possible areas for improvement, patient and staff education, and improving inhaler recycling. | 2 aspects: patients’ awareness of sustainable inhaler options and inhaler recycling | People (HCP); Process | HCP awareness of inhaler disposal/ recycling; Change in medical professionals’ prescribing practices to more sustainable inhalers |
| D'Ancona 2021 | To understand UK asthma patient's knowledge of and attitudes towards substituting high carbon MDIs with low carbon alternatives. | Patients' awareness of sustainable inhaler options |  |  |
| D'Ancona 2022 | To seek patient opinion on changing MDIs to DPIs due to their lower global warming potential. | 2 aspects: Patients' awareness of sustainable inhaler options; Patients' awareness of inhaler recycling |  |  |
| Florman 2022 | To determine the knowledge and attitudes of patients to the global warming impact of inhalers | 2 aspects: Patients' awareness of sustainable inhaler options; Patients' awareness of inhaler recycling |  |  |
| Fullwood 2022 | To prospectively evaluate the knowledge of children and families in identifying the amount of medications left in their inhalers. To review the dispital of inhalers, the information available on the NHS website, British Thoracic Society/ Scottish Intercollegiate Guidelines Network and NICE guidelines for asthma management and drug information leaflets of the commonly used inhalers. | Patients' awareness of inhaler recycling |  |  |
| Herrera 2024 | To identify patients views and behaviours related to environmental sustainability aspects of their use of inhalers for the management of asthma | 2 aspects: Patients' awareness of sustainable inhaler options; Patients' awareness of inhaler recycling |  |  |
| Khan 2021 | To describe the spectrum of manufacturer recommendations for the disposal of inhaler products in Canada. | Patients' awareness of inhaler recycling |  |  |
| Liatsikos 2021 | To identify available recycling schemes nationally and regionally and explore factors influencing availability. Subsequently, to promote recycling schemes and increase local uptake. | Factors influencing patient adherence to appropriate inhaler disposal |  |  |
| Murphy 2023 | Primary aim: to develop and assess the feasibility of a postal option for patients to return inhalers for recycling, as an alternative to the community pharmacy waste collection service. Secondary aims: to divert used or unwanted inhalers from domestic waste streams, to reduce the emissions into the atmosphere of GHGs contained in the pMDI cannisters, and to estimate the carbon footprint per inhaler and the resulting effects of the scheme in terms of reduced carbon emissions using predictive modelling. | 2 aspects: Factors influencing patient adherence to appropriate inhaler disposal; Patients' awareness of inhaler recycling |  |  |
| Narendran 2023 | To explore the different methods of disposal of metered dose inhaler canisters by patients/parents of children with asthma | Patients' awareness of inhaler recycling |  |  |
| Nurse 2022 | To increase environmental sustainability in paediatric asthma patients by increasing awareness of the correct disposal of MDIs. | 2 aspects: Factors influencing patient adherence to appropriate inhaler disposal; Patients' awareness of inhaler recycling |  |  |
| Quantz 2023 | To guide best practices for patient education on inhalers, this study surveyed perspective and knowledge around the environmental impacts of inhalers from outpatients in a British Columbia (BC) health region. | 2 aspects: Patients' awareness of sustainable inhaler options; Factors influencing patient adherence to appropriate inhaler disposal |  |  |
| Rojano 2019 | To assess self management behaviours in a prospective cohort of WTC rescue and recovery workers with asthma. | Factors influencing patient adherence to sustainable inhaler use (device/medication changes) |  |  |
| Rothwell 2024 | This project aimed to audit inhaler technique, patient preferences practice/ prescribing at secondary care sites to identify areas of improvement. To facilitate this, we developed and tested the usability of an inhaler audit tool | 4 aspects: Patients' awareness of sustainable inhaler options; Factors influencing patient adherence to sustainable inhaler use (device/medication changes); Factors influencing patient adherence to appropriate inhaler disposal; Patients' awareness of inhaler recycling |  |  |
| Savage 2023 | To examine the effect of multiple device types on adherence, and whether simpler inhaler regiments and/or adherence affect attitudes towards more sustainable | 2 aspects: Patients' awareness of sustainable inhaler options; Factors influencing patient adherence to sustainable inhaler use (device/medication changes) |  |  |
| Valero 2017 | To assess the impact of the ease of use of inhalers on adherence to treatment and on the control of symptoms | Factors influencing patient adherence to sustainable inhaler use (device/medication changes) |  |  |
| Wilkinson 2022 | To assess patient's understanding of the carbon footprint of inhalers. | Patients' awareness of sustainable inhaler options |  |  |
| **People: HCP n=3 studies (primary)** | | | | |
| Bosnic-Anticevich 2023 | To understand the extent to which healthcare professionals consider climate when selecting inhalers for patients. | Healthcare professionals' awareness of sustainable inhaler options |  |  |
| Stanley 2023 | To determine MDI prescribing prevalence as well as healthcare professionals' knowledge regarding their environmental impact | Healthcare professionals' awareness of sustainable inhaler options | Process | Change in medical professionals’ prescribing practices to more sustainable inhalers |
| Walpole 2021 | To explore prescriber knowledge and confidence on key aspects of inhaled therapies, including inhaler constituents, inhaler technique and environmental impacts of inhalers. | Healthcare professionals' awareness of sustainable inhaler options |  |  |
| **Process n=4 studies (primary) n=5 (secondary)** | | | | |
| Crooks 2024 | To analyse SABA and ICS prescribing in England during the 12-months before and after introduction of the 2022/ 2023 IIF incentives and explore the impact of SENTINEL Plus implementation in early adopter sites in England. To compare the differential prescribing changes between SENTINEL Plus early adopter sites with and without evidence of having also undertaken a switch programme in favour of lower carbon SABA inhalers. | Change in medical professionals’ prescribing practices to more sustainable inhalers |  |  |
| Galffy 2019 | To study clinical effectiveness and patient satisfaction among patients with asthma and COPD switching from MDI to Easyhaler DPI treatment in a real-life setting. | Effect of switching inhalers on respiratory health status |  |  |
| Janson 2020 | This study aimed to compare the environmental impact of DPI and MDI combinations using calculated carbon footprint data for two DPIs, Ellipta and Accuhaler, and one MDI, Evohaler. A secondary aim was to compare the inhaler-related carbon footprint impact between England and Sweden and the potential for reduction of annual carbon footprint (CO2e) in England if the pattern of inhalation devices chosen in England were to resemble that in Sweden. | Change in medical professionals’ prescribing practices to more sustainable inhalers | Product | Carbon footprint associated with different inhalers |
| Janson 2023 | To perform a post-hoc analysis on clinical outcomes on clinical outcomes data from a 12 week non-interventional single arm study. | Effect of switching inhalers on respiratory health status | Product | Carbon footprint associated with different inhalers |
| Nagasaki 2023 | To calculate inhaler usage and associated GHG emissions in Japan and evaluate the environmental and economic implications of various reduction scenarios, including the replacement of pMDI with DPI, selective replacement of DPI only for adults who can use them appropriately, and implementation of the most eco-friendly pMDI. | Change in medical professionals’ prescribing practices to more sustainable inhalers | Product | Carbon footprint associated with different inhalers |
| Nagel 2023 | To demonstrate that improved patient care could be achieved while minimizing the potential inhaler carbon footprint by comparing modelled lung delivery of inhaled corticosteroid (ICS) medications via a valved spacer. | Effect of switching inhalers on respiratory health status | Product | Carbon footprint associated with different inhalers |
| Wilkinson 2021 | To analyse the volume of SABA use and its GHG emissions versus total inhaler devices and compared the U.K with other European countries. Also calculated the annual volume and GHG emissions from SABA overuse in asthma in the U.K. using the SABINA U.K. study data. | Change in medical professionals’ prescribing practices to more sustainable inhalers | Product | Carbon footprint associated with different inhalers |
| Woodcock 2022 | To evaluate the effects of patients switching maintenance therapy from a pMDI to a DPI compared with those who continued pMDI-based treatment according to usual care on carbon footprint and asthma control | Effect of switching inhalers on respiratory health status | Product | Carbon footprint associated with different inhalers |
| Yiu 2023 | To assess if patient education sessions in a London Primary Care Network improve the sustainability and clinical outcomes of asthma care. | Effect of switching inhalers on respiratory health status |  |  |
| **Product n=9 (primary) n=23 (secondary)** | | | | |
| Alzaabi 2023 | To retrospectively quantify the carbon footprint of SABA and controller inhalers across all respiratory indications and SABA overise in asthma in LMICs, upper middle income countries and high-income countries. | Carbon footprint associated with different inhalers |  |  |
| Aumonier 2021 | To complete cradle-to-grave carbon footprint studies of two Breezehaler inhaled combinations: one containing indacaterol acetate and mometasone furoate, and one containing indacaterol acetate, mometosone furoate and glycopyrronium bromide | Carbon footprint associated with different inhalers |  |  |
| Aumonier 2022 | To determine the cradle to grave carbon footprint of two Breezhaler inhaled combinations (indacaterol acetate/ mometasone furoate and indacaterol acetate/ mometasone furoate/ glycopurronium bromide). | Carbon footprint associated with different inhalers |  |  |
| Beeh 2021 | To analyze the effect of once-daily indacaterol/ glycopyrronium/ mometasone furoate vs twice daily salmeterol/ fluticasone on hospital visits and rescue medication use in the IRIDIUM study, and its impact on carbon footprint. | Carbon footprint associated with different inhalers |  |  |
| Beeh 2021 | To report the estimate carbon footprint of managing severe exacerbations as a basis for establishing net benefits of using low carbon footprint inhalers such as BreezhalerÂ® dry powder inhaler (DPI) | Carbon footprint associated with different inhalers |  |  |
| Bell 2022 | To model GHG emissions of MART vs Mx (maintenance) +SABA regimens. | Carbon footprint associated with different inhalers |  |  |
| Borenius 2021 | To conduct a cradle to grave CO2 footprint analysis and life cycle assessment of four different Easyhaler products available for the treatment of asthma and COPD (budesonide-formoterol; salmeterol-fluticasone; salbutamol, and formoterol). | Carbon footprint associated with different inhalers |  |  |
| Choubey 2022 | To estimate the reduction in carbon dioxide equivalent (CO2e) emissions related to once-daily indacaterol acetate/mometasone furoate (IND/MF) and once-daily indacaterol acetate/mometasone furoate/glycopyrronium bromide (IND/GLY/MF), delivered using BreezhalerÂ® DPI device, compared to existing inhalers in the UK and Italy | Carbon footprint associated with different inhalers |  |  |
| Fulford 2021 | To evaluate 6 environmental impact categories (global warming potential, acidification, ozone depletion, use of resources, minerals and metals, eco-toxicity and freshwater use) of the Breezhaler in group geographies to identify major hotspots in the life-cycle of the device and to provide realistic solutions to these problems. | Carbon footprint associated with different inhalers |  |  |
| Gagne 2023 | To calculate the magnitude of the annual GHG emission reductions that could be achieved by bridging three existing practice gaps in Canada (misdiagnosis, suboptimal disease control, prescription patterns favouring high GHG-emitting options). | Carbon footprint associated with different inhalers |  |  |
| Goulet 2017 | To perform a compatative life cycle assessment of an inhaler and nebulizer system. | Carbon footprint associated with different inhalers |  |  |
| HÃ¤nsel 2019 | To compare the product carbon footprint of the propellant-free Respimat device with the FHA pMDIs, to understand the areas of the products' life cycles with the highest climate change impact and to determine the potential benefits of a reusable Respimat product. | Carbon footprint associated with different inhalers |  |  |
| Hatter 2024 | Primary aim was to to explore whether as-needed budesonide-formoterol DPI is associated with a lower carbon footprint than as-needed salbutamol pMDI or maintenance budesonide DPI plus as-needed salbutamol pMDI in adults with mild asthma using data from the Novel START trial. Secondary aims are to explore the effect of baseline asthma symptom control and the impact of adherence (budesonide maintenance aem only) on carbon footprint. | Carbon footprint associated with different inhalers |  |  |
| Haughney 2023 | To determine whether people with self-reported poor asthma control (RCP 3 questions) contributed a different carbon footprint to those with better control. | Carbon footprint associated with different inhalers |  |  |
| Hunt 2021 | Primary outcome to calculate the carbon footprint of the two treatment arms- a single combined DPI (fluticasone furoate/ vilanterol) against usual care across a 12 month period. Secondary outcomes to calculate the carbon footprint of healthcare visits and hospital stays. | Carbon footprint associated with different inhalers |  |  |
| Inget 2023 | To report results of three consecutives life cycles assessments performed with identical analyses, making them comparable for the Easyhaler portfolio. | Carbon footprint associated with different inhalers |  |  |
| Janson 2021 | To assess the greenhouse gas (GHG) emissions for (i) SABA vs total inhaler use (all respiratory indications) in 20 European countries and in Canada, and (ii) SABA overuse (prescription/dispensing of â‰¥3 canisters/year) in asthma in five European countries and two Canadian provinces (Alberta and Nova Scotia) from the SABA use IN Asthma (SABINA) programme | Carbon footprint associated with different inhalers |  |  |
| Janson 2022 | To assess the carbon footprint of hypothetically replacing DPIs or pMDIs with reusable SMI device (RespimatÂ®) | Carbon footprint associated with different inhalers |  |  |
| Janson 2023 | To compare the carbon footprint, measured in terms of CO2e emissions, of switching the COPD patient population to the SMI, Respimat Reusable. The main analysis focused on switching within the same therapeutic class, and additional scenarios were conducted to complement the main analysis. | Carbon footprint associated with different inhalers | Process | Effect of switching inhalers on respiratory health status |
| Jeswani 2019 | To evaluate a range of life cycle environmental impacts of pMDIs and DPIs. | 2 aspects: Carbon footprint associated with different inhalers ; Amount of propellants generated throughout the lifecycle of inhalers |  |  |
| Jeswani 2020 | To compare the environmental impacts of the use and end-of-life stages of these two types of inhaler device. | 2 aspects: Carbon footprint associated with different inhalers ; Amount of propellants generated throughout the lifecycle of inhalers |  |  |
| Kponee-Shovein 2022 | To 1) identify key GHG emission drivers amid asthma exacerbation care by severity level, 2) quantify the GHG emissions associated with these drivers and 3) calculate the total GHG emissions and associated costs among UK adults with asthma by exacerbation severity level. | Carbon footprint associated with different inhalers |  |  |
| Kponee-Shovein 2022 | To quantify the global climate impacts of long-term status quo asthma inhaler use and estimates the long-term global CO2e emissions and cost impacts of substituting currently available MDIs with DPIs. | Carbon footprint associated with different inhalers |  |  |
| OrtsÃ¤ter 2019 | To perform a budget impact analysis that incorporates the ecological impact of substituting Respimat disposable with RESPIMAT re-usable in the healthcare system in the Nordics (Denmark, Iceland, Finland, Norway and Sweden) and Benelux. | Carbon footprint associated with different inhalers |  |  |
| OrtsÃ¤ter 2020 | To perform an economic evaluation that incorporates the ecological impact of adopting RESPIMAT re-usable into the healthcare system in Germany. | Carbon footprint associated with different inhalers |  |  |
| Panigone 2020 | To quantify the carbon footprint of specific products using an innovative approach, as well as estimating the carbon footprint of the same products when manufactured witht he new low GWP propellant HFA152a. | Carbon footprint associated with different inhalers |  |  |
| Pernigotti 2021 | To conduct a series of scenario analyses using asthma and COPD inhaler sales data from 2019 to model emissions reductions over a 10-year period for five reference markets: the UK, Italy, France, Germany and Spain. | Carbon footprint associated with different inhalers |  |  |
| Sosnowski 2023 | To determine and compare the propellant content and emissions of 6 pMDIs commonly prescribed in Poland. | 2 aspects: Carbon footprint associated with different inhalers ; Amount of propellants generated throughout the lifecycle of inhalers |  |  |
| TenHave 2022 | To calculate the environmental impact of changing pMDI to more eco-friendly NPI change in Dutch primary and secondary respiratory healthcare and to analyse the associated pharmaceutical and device costs. | Carbon footprint associated with different inhalers |  |  |
| Varghese 2020 | To perform an environmental â€˜budgetâ€™ impact analysis that incorporates the ecological impact of substituting RESPIMAT disposable with RESPIMAT re-usable in South-East Asia and South Korea. | Carbon footprint associated with different inhalers |  |  |
| Vartiainen 2021 | To conduct a full LCA of all 6 Easyhaler products . | Carbon footprint associated with different inhalers |  |  |
| Wilkinson 2019 | To investigate a variety of scenarios for altered inhaler prescription patterns in England, and the cost implications of switching to MDIs. | Carbon footprint associated with different inhalers |  |  |
