# Supplementary material for: Inhaler sustainability in asthma and COPD care: a systematic review
Source: BMJ Open. 2025 Jul 25;15(7):e098052. doi: 10.1136/bmjopen-2024-098052 (PMC12306229; doi:10.1136/bmjopen-2024-098052)
Supplement: Supplementary file 5 [file bmjopen-2024-098052.reviewer_comments.pdf]

PEER REVIEW HISTORY

BMJ Open publishes all reviews undertaken for accepted manuscripts. Reviewers are asked to complete a checklist review form and are provided with free text boxes to elaborate on their assessment. These free text comments are reproduced below.

ARTICLE DETAILS

Title (Provisional)

A Systematic Review of Inhaler Sustainability in Asthma and COPD Care

Authors

Onasanya, Adeola Ayodotun; Haider, Yaser; Peaston, Grace; Ignatowicz, Agnieszka; Turner, Alice M

VERSION 1 - REVIEW

**Reviewer**	**1**
**Name**	**Yang, Ian**
**Affiliation**	**The Prince Charles Hospital, Metro North Hospital and Health Service**
**Date**	**17-May-2025**
**COI**	**None**

Thank you for inviting me to review this interesting paper.

Improving sustainability in relation to inhaled medicines is an important goal.

This systematic review addressed both patient and clinician perspectives, and also product/technology features. Strengths of the study include a detailed search and careful narrative descriptions of key findings.

Major comments:

1 Abstract (Page 1):

The Abstract Results could be expanded to include more of the themes and results found. It may be useful to use the same framework used in the paper (people, process, product) here in the Abstract Results, to introduce this concept, and describe the main results found in the same order as presented in the Results section of the paper. The current Abstract Results section appears quite short, and should be expanded further to capture the main themes described in the Results section of the paper. It is not clear if there is a missing section to the Abstract Results, as the last sentence might be incomplete (‘Only 31 studies demonstrated sufficiently rigorous data analysis – no full stop inserted).

2 Selection of papers (Page 4, line 41):

Was there a specific reason to limit to studies in the last 10 years? The reason should be stated.

3 Quality assessment tool (Page 5, line 8):

A new quality assessment tool was devised for this systematic review. Did the tool effectively capture different types of bias in the range of studies selected?

4 Patient perspectives (Page 9):

Did the studies cover any adverse effects of changing from pMDI plus spacer to DPI for inhaled steroids, such as increased potential risk of oral candidiasis with some DPI products, or suboptimal inhaler technique if insufficient inspiratory flow for DPI was present?

5 Product outcome (Page 11):

Were there any quantitative results from the product studies that could be pooled in a meta-analysis?

6 DPI (Page 13, line 60):

The general recommendation is to convert pMDI to DPI. Will new propellants with low carbon footprint, being developed and tested, change this recommendation? A comment in the discussion could be added.

7 Environmental impact (Page 14, line 8):

'DPIs and SMIs have a significantly lower environmental impact than pMDIs' – it is noted that the quantitative results are described in narrative form in the text of the Results section. As per query 4 above, would it be possible to combine any of the similar results/study designs to include a forest plot showing the 'significantly lower' impact?

**Reviewer**	**2**
**Name**	**Blakey, John**
**Affiliation**	**Curtin University, Medical School**
**Date**	**02-Jun-2025**
**COI**	**None**

Onasanya and colleagues present a systematic review and narrative synthesis covering three linked topics: user attitudes towards the environmental impact of inhaled medicines, HCP attitudes to the same, and the type and magnitude of the environmental impact. This is an ambitious undertaking requiring information from a variety of types of research, and thus the grading of contributory information and its presentation presents challenges. The topic is

one of great importance, and of broad interest to respiratory clinicians and researchers, as well as policy makers. The paper is largely well-written, though there are minor formatting problems and in particularly the figures need work (see below)

However, the main limitation of this paper is that it seems to bite off more than it can chew within the length of a research article, and seems more suitable to a longer report where findings and issues could be discussed fully. It doesn't comprehensively address the three questions it poses, and doesn't persuasively combine the findings into the "deeper understanding" it promises. The study also "aims to identify actionable recommendations" but doesn't really provide these either. I was left with a sense the authors have done a lot of hard work retrieving studies, and the topic is very important, but I didn't feel I'd been exposed to a new insight or key practice recommendation.

Methods

It was unclear why the authors felt studies that were not in English or were written ten years or more ago would be non-contributory. This is not discussed as a limitation in the discussion.

I note a key inclusion criteria (supp2) of an intervention related to sustainability. It would follow that a study specifically looking at the attitudes of patients to inhaler sustainability would be excluded. Seemingly either this inclusion criteria was not applied rigorously, or contributory studies were missed.

The outcomes listed in that appendix also include a couple of grey areas eg "Measured by adherence rates, medication usage logs, and patient self-reports ETC".

Also in Supp2 I assumed the authors meant "CO2 equivalents per kilogram" to be how many kg of carbon dioxide emissions warm the climate equally as 1 kg of the propellant in question. However in the text there are various "equivalents" stated related to overall use, puffs, etc so I'm not sure what the standard way of documenting it from each paper was.

Results

The PRISMA flowchart has some missing numbers, and a partially deleted line, and it seems the numbers might not be correct eg "Studies sought for retrieval",

I would have preferred the authors to be more specific with their synthesis rather than use terms like "in many studies" or "common concerns" or "mainly centred around".

Figure 3 is confusing: there are 27 bars on the chart and 14 labels. I think it needs completely redoing.

It would have been helpful to have Figure 4 organised in the same way as the text (People, Process, Product).

Figure 4 also includes the descriptor “People Patients”, which is one of a few minor errors that need to be buffed up through the manuscript. Others include using different abbreviations for metered dose inhaler, repeatedly defining DPI, and inconsistent capitalisation in the references

As noted above, trying to squeeze in a very large amount of different studies seems to result in only very topline information being provided without any synthesis. eg I don’t know what “The pre-switch adherence rate was 47.5% (with 45 out of 50 patients adhering)” means without reading the paper in question.

Relatedly, the Product section is not very instructive. Various metrics are discussed and the influence of different propellants and propellant volumes is not clear. By way of a further example, I don’t think a reader would know this statement only relates to one type of DPI from one manufacturer: “The carbon footprint for LABAs like Formeterol has been gradually decreasing, with Inget (2023) showing a reduction from 573.69 kg CO2e (2019) to 511.32 kg CO2e (2023).”

The hugely impactful mandate by EU legislation that current propellants will be almost entirely phased out by 2030 doesn’t get discussed, nor is there informative mention of new propellants that several major inhaler manufacturers have been publishing on and either have, or will roll out in routine products very soon.

Again, I appreciate a lot of hard work has gone in here and I hope an improved version of the paper that will better engage readers will come out of this process.

VERSION 1 - AUTHOR RESPONSE

Reviewer: 1

Major comments:

1. Abstract (Page 1):

The Abstract Results could be expanded to include more of the themes and results found. It may be useful to use the same framework used in the paper (people, process, product) here in the Abstract Results, to introduce this concept, and describe the main results found in the same order as presented in the Results section of the paper. The current Abstract Results section appears quite short, and should be expanded further to capture the main themes described in the Results section of the paper. It is not clear if there is a missing section to the Abstract Results, as the last sentence might be incomplete (‘Only 31 studies demonstrated sufficiently rigorous data analysis – no full stop inserted).

Response: Thank you for your comment. We agree that the Abstract Results section was overly concise and did not fully reflect the structure and depth of the main findings. We have now revised the full Abstract, including the results section, to capture the main findings. The statement cited has been moved to the results section within the main manuscript. The abstract results now read 'Patients demonstrated varying awareness and behaviours toward sustainable inhaler usage, adherence, and disposal, highlighting a need for improved education on inhaler use and disposal guidance. Healthcare professionals showed differing levels of confidence and behaviours regarding prescribing sustainable inhalers, indicating a clear need for professional training and supportive resources. Transitioning from pressurised metered-dose inhalers (pMDIs) to dry powder inhalers (DPIs) consistently demonstrated positive environmental outcomes and clinical benefits.'

2 Selection of papers (Page 4, line 41):
Was there a specific reason to limit to studies in the last 10 years? The reason should be stated.

Response: Yes, the review was limited to studies published in the last 10 years to align with major developments in global climate and health policy. Specifically, the 2014 IPCC Synthesis Report and the UK Sustainable Development Strategy identified healthcare as both a contributor to climate change and increasingly vulnerable to its impacts. Limiting the review to this period ensured the inclusion of studies reflecting approaches to environmental sustainability in healthcare. An explanatory statement has been added to the study selection criteria to reflect this view.

3 Quality assessment tool (Page 5, line 8):
A new quality assessment tool was devised for this systematic review. Did the tool effectively capture different types of bias in the range of studies selected?

Response: Yes, the devised quality assessment tool was designed to address the methodological diversity of the included studies, which ranged from mixed methods research and observational designs to environmental life-cycle assessments. It was derived from validated tools assessing the most common study designs included in the review, so that it

would capture all necessary data, whilst also standardising within the review. The tool focused on core domains applicable across study types: methodological clarity, completeness of outcome reporting, and potential for selective reporting. While it was not a validated instrument, it enabled a consistent and pragmatic evaluation of bias and quality across heterogeneous evidence types. The tool's application revealed common limitations such as incomplete data reporting.

4 Patient perspectives (Page 9):
Did the studies cover any adverse effects of changing from pMDI plus spacer to DPI for inhaled steroids, such as increased potential risk of oral candidiasis with some DPI products, or suboptimal inhaler technique if insufficient inspiratory flow for DPI was present?

Response: Thank you for this comment. No, the included studies did not systematically report on clinical adverse effects associated with switching from pMDIs (plus spacer) to DPIs, such as the risk of oral candidiasis with inhaled corticosteroids or the implications of suboptimal inspiratory flow in patients using DPIs. While some studies addressed patient concerns around inhaler effectiveness and usability, none provided objective clinical data on technique-related efficacy or adverse event rates following device transition. This represents a notable gap in the current evidence base, particularly in the context of sustainability-driven prescribing changes, and underscores the need for further research.

5 Product outcome (Page 11):
Were there any quantitative results from the product studies that could be pooled in a meta-analysis?

Response: Unfortunately, it was not possible to pool the product studies into a meta-analysis for several reasons related to methodological heterogeneity. First, the included studies differed in their definitions of what aspects of the lifecycle of the inhaler they were focused on (e.g., whether emissions from manufacturing, distribution, use, and disposal phases were included). Second, functional units of measurements differed across studies, with some studies reporting product use by device type, medication type, dosage type per year or patient use, and assumptions regarding patient behaviour and end-of-life disposal. Third, the emission values were derived from varying sources, including manufacturers, modelled

estimates, and life-cycle databases, limiting standardisation. Given these variabilities, a meta-analytic synthesis was not methodologically appropriate. Instead, the findings were narratively synthesised and organised by medication class and device type to highlight patterns and differences in carbon footprint across inhaler products.

6 DPI (Page 13, line 60):
The general recommendation is to convert pMDI to DPI. Will new propellants with a low carbon footprint, being developed and tested, change this recommendation? A comment in the discussion could be added.

Response: Thank you for this comment. While current guidance supports switching from pMDIs to DPIs due to their lower carbon footprint, the development of low-GWP propellants (e.g. HFA-152a) may change this recommendation as they become commercially viable. Future environmental comparisons will need to account not only for propellant emissions but also for patient adherence and correct inhaler use, both of which significantly influence treatment effectiveness and overall environmental impact. The above explanation has been added to the DPI section of the discussion.

7 Environmental impact (Page 14, line 8):
'DPIs and SMIs have a significantly lower environmental impact than pMDIs' – it is noted that the quantitative results are described in narrative form in the text of the Results section. As per query 4 above, would it be possible to combine any of the similar results/study designs to include a forest plot showing the 'significantly lower' impact?

Response: We appreciate the reviewer's suggestion. However, a forest plot was not included and is not feasible because the data from the included studies were not suitable for statistical pooling via meta-analysis or visual pooling in a forest plot due to substantial heterogeneity across the included studies. Specifically, studies differed in methodology and reporting. For instance, lifecycle stages differed with studies focused on one or more different aspects of carbon footprint assessment (e.g., whether emissions from raw material extraction, manufacturing, use, or disposal were included or excluded). There was variability on the devices and medication content assessed (e.g. some assessed specific device types or specific devices with certain medication contents). There was also considerable variability in

functional units of reporting, with emissions reported per dose, per actuation (puff), per patient-year, or per treatment course, as well as inconsistencies in carbon accounting methods and outcome reporting. Furthermore, several studies did not report confidence intervals or measures because the data was based on exact data (e.g. number of prescribed pMDIs in England). Under these conditions, a forest plot would risk oversimplifying findings that are highly context-dependent and methodologically diverse, potentially misrepresenting the comparability of results. We therefore adopted a structured narrative synthesis, which better reflects the available evidence while highlighting consistent directional trends showing the lower environmental impact of DPIs and SMIs relative to pMDIs.

Reviewer: 2

Dr. John Blakey, Curtin University, Sir Charles Gairdner Hospital

Comments to the Author:

Onasanya and colleagues present a systematic review and narrative synthesis covering three linked topics: user attitudes towards the environmental impact of inhaled medicines, HCP attitudes to the same, and the type and magnitude of the environmental impact. This is an ambitious undertaking requiring information from a variety of types of research, and thus the grading of contributory information and its presentation presents challenges. The topic is one of great importance, and of broad interest to respiratory clinicians and researchers, as well as policy makers. The paper is largely well-written, though there are minor formatting problems and in particularly the figures need work (see below)

However, the main limitation of this paper is that it seems to bite off more than it can chew within the length of a research article, and seems more suitable to a longer report where findings and issues could be discussed fully. It doesn't comprehensively address the three questions it poses, and doesn't persuasively combine the findings into the "deeper understanding" it promises. The study also "aims to identify actionable recommendations" but doesn't really provide these either. I was left with a sense the authors have done a lot of hard work retrieving studies, and the topic is very important, but I didn't feel I'd been exposed to a new insight or key practice recommendation.

Response: We appreciate the reviewer's constructive feedback, including the recognition of the scope, importance, and relevance of this topic for respiratory clinicians, researchers, and

policymakers. We acknowledge the challenge posed by synthesising evidence from three distinct but interrelated domains of people, product and process, which broadly draws on diverse study types and methodologies.

Regarding the concern that the paper attempts to cover too much within the constraints of a single research article, we had a choice in making it shorter, but this comes at the risk of losing useful insights we found from the review. However, we took several steps to improve clarity and focus based on limitations faced, including applying a framework to provide a structured lens for interpreting findings. This framework has been applied consistently in the results and discussion to clarify the connections across the three domains and to enhance conceptual understanding.

We have revised the discussion section and have explicitly identified a set of actionable recommendations (see Table 1), now presented in a dedicated summary table, to address gaps that influence sustainability outcomes. While we agree that the topic could benefit from a more extensive report or policy brief in future work, we believe the revised manuscript provides a stronger synthesis of the current evidence base and clearer insights for clinical and policy audiences within the scope of a research article. We also appreciate the reviewer's note on figures and formatting, and we have revised the figures accordingly for clarity and consistency.

Methods

It was unclear why the authors felt studies that were not in English or were written ten years or more ago would be non-contributory. This is not discussed as a limitation in the discussion. I note a key inclusion criteria (supp2) of an intervention related to sustainability. It would follow that a study specifically looking at the attitudes of patients to inhaler sustainability would be excluded. Seemingly either this inclusion criteria was not applied rigorously, or contributory studies were missed.

Response: Thank you for this insightful comment. The review was limited to English-language publications due to resource constraints and the lack of capacity for translation or appraisal of non-English manuscripts. This limitation is now acknowledged under the strengths and weaknesses section. The decision to limit studies published from 2014 onward was based on relevance to recent interest in sustainability in healthcare. The year 2014 was selected as a reference point due to the release of the Intergovernmental Panel on Climate

Change (IPCC) Synthesis Report and the UK NHS Sustainability strategy document, which showed a shift in awareness of the interrelationship between healthcare and sustainability. Studies published after this point are more likely to reflect evolving sustainability interests, particularly with the inhaler, which contributes 3% of the NHS greenhouse gas emissions. We have provided a clearer justification in the methods section (study selection criteria).

Regarding the use of 'intervention', we recognise that the original use of the term "intervention" in our inclusion criteria may have been misleading, as many of the included studies were observational or descriptive. To address this, we have revised our conceptual approach using the PEO (Population, Exposure, Outcome) framework, which better reflects the scope of the included literature. In this context, 'exposure' refers broadly to sustainability-related aspects of inhaler use such as awareness, attitudes, prescribing behaviours, device selection, disposal behaviours, and recycling, regardless of whether a formal intervention was evaluated. These revisions have been made in supplementary file 2 to reflect our intention.

The outcomes listed in that appendix also include a couple of grey areas eg "Measured by adherence rates, medication usage logs, and patient self-reports, ETC".
Also in Supp2 I assumed the authors meant "CO2 equivalents per kilogram" to be how many kg of carbon dioxide emissions warm the climate equally as 1 kg of the propellant in question. However in the text there are various "equivalents" stated related to overall use, puffs, etc so I'm not sure what the standard way of documenting it from each paper was.

Response: Thank you for your observation and comment. We acknowledge the lack of precision in some outcome descriptions in Supplementary file 2 and have revised the phrasing to remove vague terms. We now specify the exact outcome measures used as 'adherence rates from prescription refill data, electronic medication monitoring data, or self-report tools.'

Regarding the carbon emissions, indeed, CO_2 equivalents per kilogram (kg CO_2e) were reported inconsistently across studies e.g. per dose, per inhaler, per actuation, per year of use, or per treatment course. This is because CO2 equivalents must be contextualised to reflect the data input. For instance, calculating the CO2 equivalent of a dose of a specific inhaler would be recorded as 1.5 CO2e/kg per dose, giving more context to the meaning of the data. We reported the original units used in each study and avoided aggregation and conversions across

non-comparable functional units. We also included a note on this heterogeneity as a limitation of our synthesis.

Results

The PRISMA flowchart has some missing numbers, and a partially deleted line, and it seems the numbers might not be correct e.g. “Studies sought for retrieval”,

Response:

Thank you for highlighting this. We have reviewed and corrected the PRISMA flowchart to ensure all required fields are completed accurately, including restoring the partially deleted line and verifying the consistency of reported numbers.

I would have preferred the authors to be more specific with their synthesis rather than use terms like “in many studies” or “common concerns” or “mainly centred around”.
Figure 3 is confusing: there are 27 bars on the chart and 14 labels. I think it needs completely redoing.

Response: Thank you for this helpful feedback. We have revised the manuscript to improve specificity in the narrative synthesis by replacing vague terms such as “in many studies” and “common concerns” with more precise words.

Regarding Figure 3, we have now redesigned the figure to ensure consistent labelling and clearer visual grouping of categories. .

It would have been helpful to have Figure 4 organised in the same way as the text (People, Process, Product).
Figure 4 also includes the descriptor “People Patients”, which is one of a few minor errors that need to be buffed up through the manuscript. Others include using different abbreviations for metered dose inhaler, repeatedly defining DPI, and inconsistent capitalisation in the references

Response: Thank you for your detailed and constructive feedback. We have revised Figure 4 to align its structure with the People–Process–Product (PPP) framework used in the text, including removing the people-patient text.

We also made changes to the manuscript to address the remaining editorial inconsistencies. This includes standardising abbreviations (e.g. consistently using ‘pMDI’ for pressurised metered dose inhaler), avoiding repeated definitions of DPI, and correcting inconsistencies in reference formatting and capitalisation. These changes have been incorporated into the revised version.

As noted above, trying to squeeze in a very large amount of different studies seems to result in only very topline information being provided without any synthesis. eg I don’t know what “The pre-switch adherence rate was 47.5% (with 45 out of 50 patients adhering)” means without reading the paper in question.
Relatedly, the Product section is not very instructive. Various metrics are discussed and the influence of different propellants and propellant volumes is not clear. By way of a further example, I don’t think a reader would know this statement only relates to one type of DPI from one manufacturer: “The carbon footprint for LABAs like Formeterol has been gradually decreasing, with Inget (2023) showing a reduction from 573.69 kg CO2e (2019) to 511.32 kg CO2e (2023).”

Response: Thank you for this valuable feedback. We acknowledge that the breadth of included studies led to some sections offering only surface-level findings without sufficient interpretive synthesis. In response, we have revised the results and discussion sections, particularly the Product domain to provide clearer context for reported figures. For instance, some context has been added for the ‘pre-adherence rate’ by rephrasing as ‘The pre-switch adherence rate for previously prescribed inhalers was 47.5% (with 45 out of 50 patients adhering), but post-switch adherence rates for current inhalers were not provided.’

We now clarify that the LABA carbon footprint figure cited from Inget (2023) refers to a gradual reduction in carbon footprint over 5 years. An explanatory text has been added to product outcome to explain the variation in carbon footprint reporting style. It reads as ‘Reporting methods varied, with carbon footprints expressed per actuation, medication dose, device, or annually, depending on each study’s objectives.’ Under the product outcome.’

The hugely impactful mandate by EU legislation that current propellants will be almost

entirely phased out by 2030 doesn't get discussed, nor is there informative mention of new propellants that several major inhaler manufacturers have been publishing on and either have, or will roll out in routine products very soon.

Again, I appreciate a lot of hard work has gone in here and I hope an improved version of the paper that will better engage readers will come out of this process.

Response: Thank you for this insightful comment. We agree that the impending phase-out of high-GWP propellants under EU legislation represents a major shift in inhaler sustainability and should be more prominently discussed. We have updated the Discussion section to acknowledge this regulatory change, referencing the European F-gas regulations that will lead to the near-total phase-out of current propellants by 2030, the launch of ewer propellants (HFA-152a), and noted how these innovations would need to be assessed for their environmental impact in future compared with DPIs and SMIs**.**
